# Implementation of a Health-System Wide Antimicrobial Stewardship Program in Omaha, NE

**DOI:** 10.3390/pharmacy7040156

**Published:** 2019-11-25

**Authors:** Jennifer Anthone, Dayla Boldt, Bryan Alexander, Cassara Carroll, Sumaya Ased, David Schmidt, Renuga Vivekanandan, Christopher J. Destache

**Affiliations:** 1 Department of Pharmacy Services, CHI Health, Omaha, NE 68124, USA; jennifer.anthone@alegent.org (J.A.); Dayla.boldt2@alegent.org (D.B.); Bryan.alexander@alegent.org (B.A.); Cassara.carroll@alegent.org (C.C.); Sumaya.ased@alegent.org (S.A.); David.Schmidt2@alegent.org (D.S.); Renugavivekanandan@creighton.edu (R.V.); 2 School of Medicine, Creighton University, Omaha, NE 68178, USA; 3 School of Pharmacy & Health Professions, Creighton University, Omaha, NE 68178, USA

**Keywords:** antimicrobial stewardship, healthcare system, pharmacy service

## Abstract

The Centers for Medicare and Medicaid Services (CMS) have mandated that acute care and critical access hospitals implement an Antimicrobial Stewardship (AMS) Program. This manuscript describes the process that was implemented to ensure CMS compliance for AMS, across a 14-member health system (eight community hospitals, five critical access hospitals, and an academic medical center) in the Omaha metro area, and surrounding cities. The addition of the AMS program to the 14-member health system increased personnel, with a 0.5 full-time equivalent (FTE) infectious diseases (ID) physician, and 2.5 FTE infectious diseases trained clinical pharmacists to support daily AMS activities. Clinical decision support software had previously been implemented across the health system, which was also key to the success of the program. Overall, in its first year, the AMS program demonstrated a $1.2 million normalized reduction (21% total reduction in antimicrobial purchases) in antimicrobial expenses. The ability to review charts daily for antimicrobial optimization with ID pharmacist and physician support, identify facility specific needs and opportunities, and to collect available data endpoints to determine program effectiveness helped to ensure the success of the program.

## 1. Health-System Background

CHI Health, a 14-hospital health system in Nebraska and southwestern Iowa, is comprised of one academic medical center (~300 beds), eight acute care community hospitals, ranging in average daily census of 10–200 patients each, and five critical access hospitals (25 beds or less). Services offered within the diverse health-system include a Level 1 trauma center; regional burn unit, Level 3 NICU, specialty cardiac surgery hospital, specialty spine orthopedic hospital, and two behavioral health facilities. The system spans urban, suburban, and rural settings with a combined average daily census of 900–1000 patients. Of the hospitals, five are located in a close geographical footprint, historically shared policies, procedures, and medical staff bylaws, while the four more distant community hospitals, and five critical access hospitals, operated independently. Within the health-system, nine facilities shared a common instance of a fully integrated electronic health records (EHR) with computerized provider order entry (CPOE), and five facilities had independent medical records that did not include CPOE. The health-system had previously implemented a clinical decision support tool that allowed patients across multiple facilities to be reviewed, despite having different medical records. Infectious disease (ID) provider consultation was available daily at eight of the 14 facilities, and an ID trained pharmacist was available at one of the 14 hospitals. Health-system diversity in facility size, EHR, and ID resource availability is common within medium to large health-systems [1].

The Antimicrobial Stewardship Program deployment and effectiveness across the health system was similarly diverse. The five integrated acute care hospitals instituted a joint Antimicrobial Stewardship Program in 2010, based in part on the 2007 IDSA-SHEA Antimicrobial Stewardship (AMS) guidelines [2]. During this time, 1–2 selected hospital pharmacists from each site were asked to provide AMS functions by reviewing antimicrobial alerts daily, in addition to their other daily responsibilities. At the same time, a contract with a private ID physician was implemented. This ID physician chaired the joint Antimicrobial Stewardship Committee and was available to the identified pharmacists on an as-needed basis, to assist with guiding pharmacist recommendations. The pharmacists then communicated these recommendations to the prescribing providers. This program was implemented without any additional pharmacist resources or hours, and with minimal pharmacist training in infectious diseases.

In 2012, Creighton University Medical Center (CUMC) joined the five-hospital health-system mentioned above. CUMC, the Creighton University Health Sciences affiliated teaching institution, with a Level 1 trauma service, had implemented its own AMS program in 2011. However, this program included a dedicated pharmacist FTE responsible for all AMS activities, and a partnership with the Creighton University Infectious Disease division for ID physician support. The AMS pharmacist was responsible for daily prospective patient reviews of all CUMC hospitalized patients on antimicrobial therapy, and would review complex patient cases requiring intervention with the dedicated Creighton University ID provider on service. Recommendations to optimize antimicrobial therapy were made to the prescribing providers by the AMS pharmacist and/or Creighton University ID provider.

The remaining independent hospitals joined with the newly formed system in 2013 to form the regional health-system. These hospitals had also recently implemented independent antimicrobial stewardship programs, without additional ID trained pharmacist or ID trained provider resources.

In 2014, after President Obama signed the executive order to combat antimicrobial resistance, the Centers for Medicare/Medicaid Services (CMS) made AMS mandatory for all acute care and critical access hospitals, starting January 1, 2017. The CMS measures were based primarily on the CDC Core Elements of Antimicrobial Stewardship [3], which the health-system was not fully achieving, with historical AMS practice models. The health-system pharmacy service line and Creighton University ID division developed a workgroup to assess the current AMS practices at each of the hospitals to ensure CMS standards and CDC Core Elements were being met, and to develop a plan to address any gaps within hospitals in the health system.

## 2. Independent Antimicrobial Stewardship Models

Within the health-system, three different models for AMS practices were identified. The academic medical center (AMC) had a robust AMS program including ID trained pharmacist and ID physician support. The AMC utilized these resources to perform prospective audit and feedback for all patients receiving antimicrobials in the hospital daily. As previously discussed, the framework for this program was a daily meeting between the ID pharmacist and ID clinician who made recommendations to providers and house staff. Community hospitals all utilized non-ID trained pharmacists to monitor antimicrobials using a clinical decision support tool and medication profile review, which occurred most days, in addition to other daily pharmacist activities. Most of these facilities had access to an ID provider, or a non-ID trained provider champion, who the pharmacists could contact as needed for guidance on challenging cases, to help improve their AMS recommendations. Lastly, the critical access hospitals had non-ID trained pharmacists with limited clinical review of antimicrobials, no clinical decision support tool, and no ID physician support to aid their AMS recommendations.

## 3. Development of a Health-System Wide AMS

**Figure 1:**
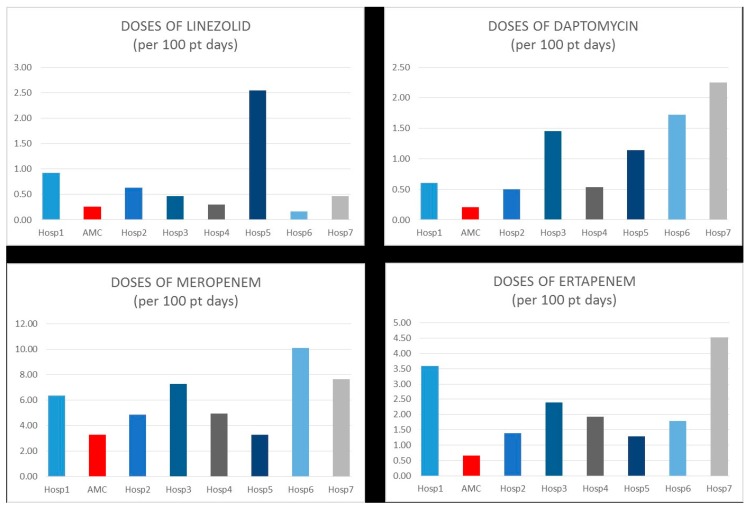
Baseline (2017) antimicrobial use (doses/100 patient days) within the health system for linezolid, daptomycin, meropenem and ertapenem.

The health-system, in preparation for the 2017 CMS requirements, found that anti-infective agents accounted for $5.6 million (~10%) of the entire health-system medication budget ($58.7 million). Analyses were performed of four broad spectrum, high cost, antimicrobials (linezolid, meropenem, ertapenem, and daptomycin) to assess current AMS effectiveness, and to identify possible opportunities for use reduction. The analysis revealed that the AMC model for AMS consistently outperformed the community hospital AMS model (Figure 1). Further supporting the success of the AMC-AMS model was that in addition to the relatively low utilization of selected broad-spectrum antimicrobials, the AMC had the highest patient acuity level of all the hospitals in the system. The critical access model was not assessed due to low patient volumes and a lack of data available for all facilities.

It was hypothesized that if the health-system could adopt the AMC process with dedicated ID-trained pharmacists and a dedicated ID physician to support daily AMS reviews, the cost savings from a reduction in high-cost, broad-spectrum anti-infective drug expenses could offset the cost of the FTE resources required. Further, the health-system could duplicate the patient outcome benefits which the AMC AMS had previously demonstrated [4]. The goal then became to attain the proper ID trained resources to expand the AMC model and processes to be the norm for the health-system [5,6].

Various reports [7,8,9] of staffing requirements for AMS programs were reviewed (Table 1). It was determined that due to geographic separation and relatively small daily patient censuses, each facility would not be able to support their own ID pharmacist and physician, as the AMC could. Alternatively, the health-system proposed a shared resources virtual monitoring model where ID pharmacists would be onsite at a minimal number of selected facilities and would monitor patients at other locations, utilizing the electronic health record and clinical decision support software. Similarly, the ID trained pharmacists would need to interface with the offsite ID physician dedicated to the AMS program.

**Figure 2:**
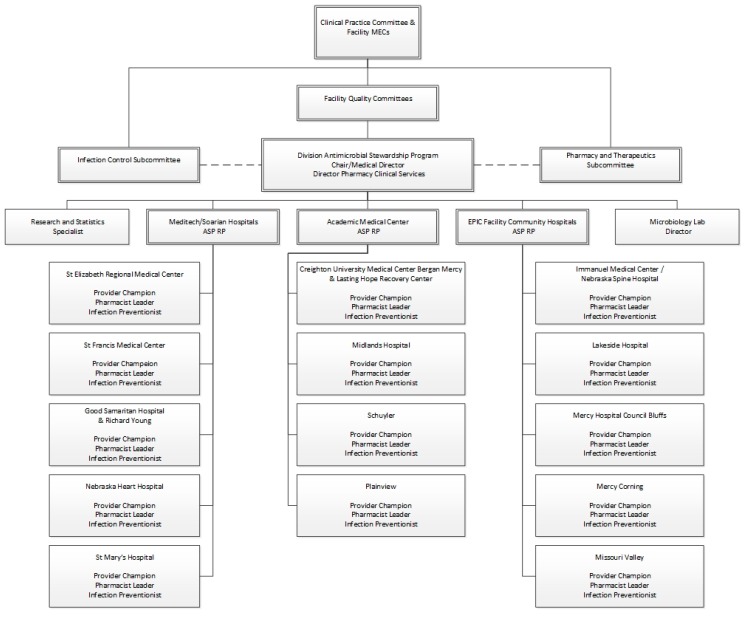
CHI Health Nebraska AMS Organizational Chart.

The Canadian AMS working group [10] concluded from review of regulatory requirements that the minimum human resources needed would include one ID physician, three ID-trained clinical pharmacists, 0.5 program administrative coordination support, and 0.4 data analyst support, as full-time equivalents (FTEs), per 1000 acute care beds. Additionally, IDSA had made recommendations for AMS staffing [11,12]. Thus, the CHI Health AMS was patterned after these recommendations and combined all facilities into one facility of ~1000 acute care hospital beds. CHI Health proposed and implemented an AMS program with a 0.5 FTE academic ID physician, 2.5 FTEs ID-trained clinical pharmacists, and a research-education component on an as-needed basis to aid with clinical research and developing educational resources for the 14-hospital health system. The 0.5 FTE ID physician would support the administration, education, and daily audit and feedback processes that made the AMC successful. The hospitals were divided by geography, size, and medical record into three groups, each managed by one of the ID trained pharmacists Figure 2.

## 4. AMS Patient Review Processes

Specific alerts requiring AMS review at each site include the following: all positive CDI results [13,14], all positive blood cultures [15,16], and any positive influenza results [17] from the hospital laboratory. Additionally, patients with multi-drug resistant organisms (including methicillin-resistant *Staphylococcus aureus* (MRSA), vancomycin-resistant *Enterococcus* (VRE), extended-spectrum beta-lactamase (ESBL), and carbapenem-resistant *Enterobacteriaceae* (CRE)) are reviewed by AMS. Lastly, patients receiving a prolonged duration of therapy [18,19,20] (>72 h), and those receiving targeted antimicrobials (i.e., appropriate use criteria, broad-spectrum, and/or high cost agents), are reviewed by AMS [21].

The AMS pharmacists use various methods to identify which patients to review—mainly utilizing clinical decision support software, which is very beneficial when covering multiple facilities. Across the division, the de-centralized clinical pharmacists manage many of the protocol-driven ASP interventions, such as the pharmacokinetic service, IV to PO transitions, renal dose adjustments, alternative dosing interchanges (for example, extended-infusion piperacillin/tazobactam), appropriate antimicrobial use criteria, and finally, those patients at high risk for *Clostridioides difficile* infection (CDI) (on antimicrobials and acid suppression therapy). This allows the AMS pharmacists to focus on the more complicated alerts such as all positive culture results (including email alerts for positive blood and other sterile site cultures), positive *Clostridioides difficile*, bug-drug mismatches, patients on broad-spectrum antimicrobials, any redundant therapy (double beta-lactams, dual anaerobic coverage, double *Pseudomonas* coverage), and those patients on three day, or greater, antimicrobial therapy to evaluate de-escalation opportunities.

Although clinical decision support software is helpful in identifying patients that may benefit from AMS review, other members of the healthcare team bring some of the most interesting and important cases up for discussion. For example, calls may come from the microbiology department about an interesting and unusual gram stain, or multi-drug resistant susceptibility requiring further testing. In addition, infection preventionists are actively monitoring patients that should be in isolation and gathering outside hospital records for patients transferring to CHI Health facilities. Lastly, with the growing success of the AMS program, providers themselves have been requesting input from the team, which is particularly common at some of the more isolated facilities that do not have an onsite infectious diseases consult service available.

Each morning, the AMS pharmacist reviews various TheraDoc reports and email alerts. Pharmacists review the history, medications, laboratory, and radiology results, as it is important to have a full picture of what is going on to be prepared for daily rounds with the ID physician. Despite being spread throughout the state in different locations, each of the AMS pharmacists has dedicated rounds Monday-Friday, either in person or via telephone. Due to the challenges of working remotely, phone calls are typically made following AMS rounds to the local pharmacist covering that patient to discuss recommendations, and to ensure the accuracy of the patient assessment. Interventions are communicated to providers in a variety of ways, such as via phone call by the AMS pharmacists or onsite pharmacist, via fax/page, and/or with a progress note placed in the patient’s chart. After a recommendation has been made, AMS pharmacists continue to follow each patient to make sure the initial intervention is still appropriate, to help with any results from diagnostic tests that had been recommended, and to assist with the duration of therapy and/or discharge arrangements with home infusion.

When tackling a division-wide AMS program and covering multiple facilities, it is very important to identify the specific needs for each site. Reaching out to local onsite colleagues to identify the biggest challenges and collaborating to work on an improvement plan has proven to be very effective. The following are examples of three different community hospitals and how AMS review processes and interventions differ: Hospital #1 has no ID consultation service available and because of this, AMS spends most of the time reviewing complicated patients that would typically have an ID physician at the bedside. It is not unusual for the AMS team to recommend transfer for higher levels of care, although this is often not feasible. In contrast, hospital #2′s biggest issue was having the highest rate of hospital-acquired CDI in the division. AMS has targeted patients with broad-spectrum antibiotics empirically to try to de-escalate sooner, and has also partnered with the infection preventionists and quality department to provide education about appropriate testing. Lastly, hospital #3 does a great job de-escalating antibiotics and targeting therapy by a 72 h time-out, but providers often continue therapy for much longer durations. AMS has focused effort on stop dates and providing literature supporting shorter durations of therapy.

Lastly, another important aim when covering multiple facilities is to understand the variability in provider types. The physician groups are going to differ greatly between the resident physicians at the AMC compared to the smaller community hospitals, but even community hospitals differ from one another. There are unique challenges in providing stewardship to private primary care providers, including the difficulty of getting ahold of them compared to hospital-employed providers.

As discussed previously, this division-wide AMS program was developed to help meet TJC requirements, which is often a challenge to accomplish, especially at smaller hospitals without as many resources. The daily activities of this unique remote AMS model meet TJC AMS requirements [22]. The leaders of the healthcare system have made AMS an organizational priority by establishing new positions and continuing to support the program. The AMS is a multidisciplinary team comprised of pharmacists, physicians, infection preventionists, and microbiologists that communicate daily and meet formally on a monthly basis. Education is given to providers in a multitude of ways, including feedback on individual patient cases and in continuing education presentations to multi-disciplinary audiences. Additionally, the health system approves multidisciplinary protocols that are generated from the AMS sub-committee of the P & T Committee. Patient data is collected, analyzed, and reports are generated. This information is used to implement new protocols and order sets, to report data from within the division as well as from National Healthcare Safety Network (NHSN), and to develop both local and divisional research, and patient improvement goals. Finally, improvements in antimicrobial use are determined at the start of each new budget year.

## 5. Overcoming Community Barriers

Local solutions that create a collective willpower have a better chance of making an impact. To accomplish this, knowledge is needed to increase awareness of problems (i.e., antimicrobial resistance) and shift attitudes. Interpersonal communication and removing barriers encourage people to adopt the new behavior, and finally, reducing threats helps people to achieve the new behavior [23]. Antimicrobials have been identified as drugs prescribed out of “fear” [24,25]. It is important to determine the root cause for overprescribing. For example, some clinicians are uncomfortable with infectious diseases, antimicrobial mechanisms, and side effects. Some clinicians are busy and have difficulty with the daily process of de-escalation or discontinuing broad-spectrum antimicrobial agents. Some clinicians reference their long clinical experience or prior institutional practices which prevents them from adopting a new behavior. However, if experts at the AMC are making an impact with stewardship, there may be benefits to following what the experts are doing. These results have been discussed at different physician forums for AMS buy-in. Finally, some are disengaged towards stewardship goals and AMS personnel. Engaging local leaders that clinicians trust has helped engage these individuals personally in order set development, protocol development and approval, and committee discussions.

Since the health system has hospitals throughout eastern and central Nebraska, as well as western Iowa, it is complicated to have local support and buy-in for AMS at all these sites. Our AMS has designated local physician and pharmacy department champions, whose role is to be active and participate in division meetings. We share ongoing antimicrobial stewardship initiatives at the local level and prepare regular reports to share with key stakeholders. This allows open communication with the local sites and allows AMS to be visible to all.

Working with the Department of Health and Human Services of Nebraska, the ID stewardship provider is a member of the statewide Hospital Acquired Infection taskforce. This taskforce is responsible for reviewing statewide epidemiology for hospital-acquired infections. Our AMS has been part of the ASP Summit as both a planning member as well as presenting at the statewide ASP symposium, impacting the entire state AMS. Finally, with grant support ($100,000) from the CDC, our AMS has been able to implement the technology for downloading antimicrobial use (AU) and antimicrobial resistance (AR) data to the CDC, to be included in their data.

## 6. Initial Findings and Impact Following AMS System Implementation

**Figure 3:**
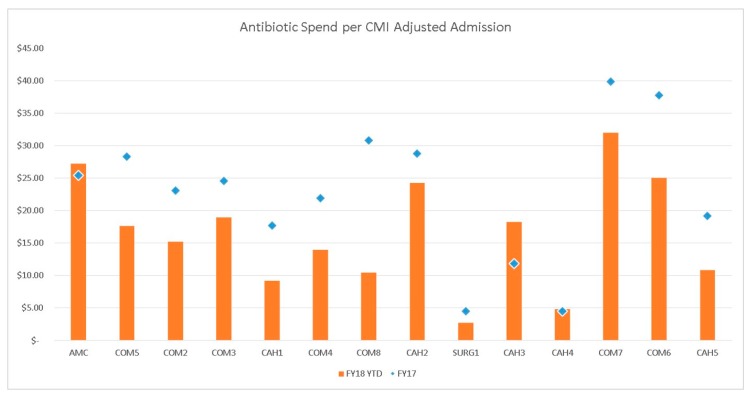
Antimicrobial drug spend per CMI adjusted admission for initial (2017) (blue diamond) and subsequent (2018) (orange bar graph) year. Abbreviations: AMC = academic medical center; COM5 = community hospital #5; COM2 = community hospital #2; COM3 = community hospital #3; CAH1 = critical access hospital #1; COM4 = community hospital #4; COM8 = community hospital #8; CAH2 = critical access hospital #2; SURG1 = surgical center #1; CAH3 = critical access hospital #3; CAH4 = critical access hospital #4; COM7 = community hospital #7; COM6 = community hospital #6; CAH5 = critical access hospital #5; FY18 = fiscal year 2018; and FY17 = fiscal year 2017.

**Figure 4:**
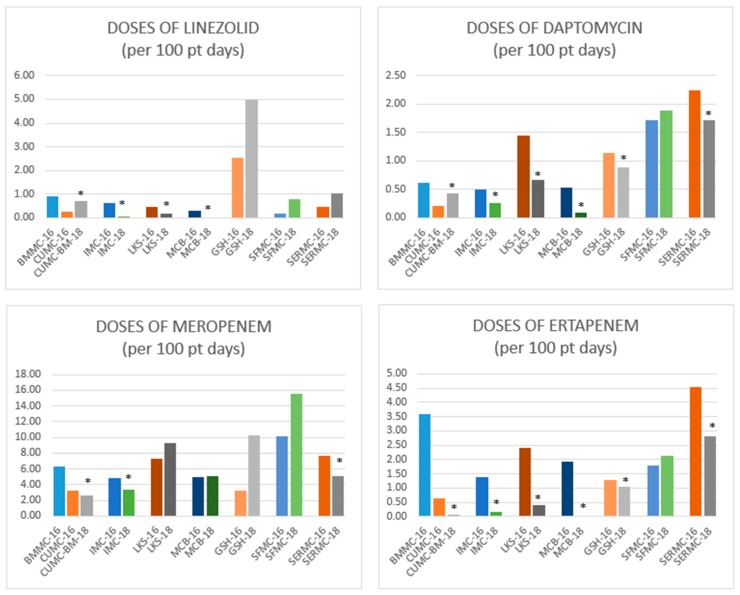
Broad-spectrum antimicrobial use before (2016) and after (2018) AMS implementation. X-axis are the different hospitals within the health system for 2016, compared to 2018. Asterisks indicate significance, *p* < 0.05. Abbreviations: BMMC = Bergan Mercy Medical Center Omaha, NE; CUMC = Creighton University Medical Center Omaha, NE; CUMC-BM = Creighton University Medical Center-Bergan Mercy Omaha, NE; IMC = Immanuel Medical Center Omaha, NE; LKS = Lakeside Hospital, Omaha, NE; MCB = Mercy Hospital in Council Bluffs, IA; GHS = Good Samaritan Hospital, Kearney, NE; SFMC = St. Francis Medical Center, Grand Island, NE; SERMC = St. Elizabeth Regional Medical Center, Lincoln, NE.

Prior to the implementation of the expanded AMS programs at CHI Health in Nebraska and western Iowa, the health system spent approximately 10% of its budget on antimicrobials. One year later, the healthcare system saw a 21% reduction (from $5.9 million to $4.4 million) in antimicrobial expenses (Figure 3), despite the overall drug budget continuing to grow ($58.7 million to $66.5 million). More exciting was improvements in patient outcomes. The AMS program produced a significant 11% reduction in 30-day readmission for *C. difficile* infection at the academic medical center. Comparison of antimicrobial usage throughout the health system demonstrated a significant reduction in linezolid, meropenem, daptomycin, and ertapenem (Figure 4). Overall, the AMS program demonstrated volume-adjusted savings of $1.2 million in its first year. Certainly, additional data will need to be gathered to determine if this trend continues.

It is important to note that these results are only from the first year after instituting the health-system AMS. Further data will need to be gathered to determine if this trend continues. Our results add to the literature showing that AMS improves antimicrobial usage, providing the right drug for the patient in the right dose, for the most appropriate duration. We believe that our AMS program could be duplicated in other health systems with similar results. Key to AMS success is the AMS physician who makes rounds (in person or by phone) with the AMS pharmacists, and is willing to contact providers for additional tests, antimicrobial duration, as well as other areas that are not recorded in this overall review of the past year. However, several of the hospitals identified in Figure 3 have not demonstrated a significant reduction in antimicrobial expenses compared to the rest of the health system. A number of reasons for this include a changing patient population, an increase in the numbers of patients with significant burn injuries (requiring antimicrobial use to increase), and/or the changing clinician demographics with increasing mid-level practitioners. Further data will need to be examined at these facilities so that we can monitor this.

## 7. Conclusions

Having dedicated AMS resources (ID-trained pharmacists and providers along with clinical decision support) deployed across multiple facilities can improve program outcomes. Clinical decision support alerts combined with prospective audit and feedback interventions can optimize patient outcomes and reduce antimicrobials expenditures. The ability to collect available data endpoints to determine program effectiveness and specific facility needs has helped to ensure program success.

## Figures and Tables

**Table 1:** Previous Antimicrobial Stewardship (AMS) staffing models.

Reference	Cooper et al. (2016)	Echevarria et al. (2017)	Nowak et al. (2012)	Trivedi et al. (2014)
Infectious Diseases provider (FTE)	0.1	-	1.0	0.56
Pharmacist provider (FTE)	0.25	1.0	3.0	1.69
Data Analysis (FTE)	0.05	-	0.4	-
Beds	~124	Per 100 beds	Per 1000 beds	Per 501–1000 beds

FTE: Full Time Equivalent.
